# Effect of *Cyrtostachys renda* Fiber Loading on the Mechanical, Morphology, and Flammability Properties of Multi-Walled Carbon Nanotubes/Phenolic Bio-Composites

**DOI:** 10.3390/nano11113049

**Published:** 2021-11-12

**Authors:** Tamil Moli Loganathan, Mohamed Thariq Hameed Sultan, Qumrul Ahsan, Mohammad Jawaid, Jesuarockiam Naveen, Ain Umaira Md Shah, Abd. Rahim Abu Talib, Adi Azriff Basri, Che Nor Aiza Jaafar

**Affiliations:** 1 Department of Aerospace Engineering, Faculty of Engineering, Universiti Putra Malaysia, UPM Serdang 43400, Selangor Darul Ehsan, Malaysia; tamilmoli@yahoo.com (T.M.L.); ainumaira91@gmail.com (A.U.M.S.); abdrahim@upm.edu.my (A.R.A.T.); adiazriff@upm.edu.my (A.A.B.); 2 Laboratory of Biocomposite Technology, Institute of Tropical Forestry and Forest Products, Universiti Putra Malaysia, UPM Serdang 43400, Selangor Darul Ehsan, Malaysia; jawaid@upm.edu.my; 3 Aerospace Malaysia Innovation Centre (944751-A), Prime Minister’s Department, MIGHT Partnership Hub, Jalan Impact, Cyberjaya 63000, Selangor Darul Ehsan, Malaysia; 4 Department of Mechanical and Production Engineering, Ahsanullah University of Science and Technology, Dhaka 1208, Bangladesh; qumrul.mpe@aust.edu; 5 School of Mechanical Engineering, Vellore Institute of Technology, Vellore 632014, India; naveen.j@vit.ac.in; 6 Department of Mechanical and Manufacturing Engineering, Universiti Putra Malaysia, UPM Serdang 43400, Selangor Darul Ehsan, Malaysia; cnaiza@upm.edu.my

**Keywords:** MWCNT, phenolic, CR fiber, mechanical properties, morphology, flammability, TOPSIS

## Abstract

This research focuses on evaluating the effect of *Cyrtostachys renda* (CR) fiber and the impact of adding multi-walled carbon nanotubes (MWCNT) on the morphological, physical, mechanical, and flammability properties of phenolic composites. MWCNT were supplemented with phenolic resin through a dry dispersion ball milling method. Composites were fabricated by incorporating CR fiber in 0.5 wt.% MWCNT-phenolic matrix by hot pressing. Nevertheless, the void content, higher water absorption, and thickness swelling increased with fiber loading to the MWCNT/phenolic composites. The presence of MWCNT in phenolic enhanced the tensile, flexural, and impact strength by as much as 18%, 8%, and 8%, respectively, compared to pristine phenolic. The addition of CR fiber, however, strengthened MWCNT-phenolic composites, improving the tensile, flexural, and impact strength by as much as 16%, 16%, and 266%, respectively, for 50 wt.% loading of CR fiber. The CR fiber may adhere properly to the matrix, indicating that there is a strong interface between fiber and MWCNT-phenolic resin. UL-94 horizontal and limiting oxygen index (LOI) results indicated that all composite materials are in the category of self-extinguishing. Based on the technique for order preference by similarity to the ideal solution (TOPSIS) technique, 50 wt.% CR fiber-reinforced MWCNT-phenolic composite was chosen as the optimal composite for mechanical and flammability properties. This bio-based eco-friendly composite has the potential to be used as an aircraft interior component.

## 1. Introduction

Over the next few decades, the aviation industry will face substantial increases in air travel, as well as the aggressive target of reducing environmental effects in compliance with stringent European regulations. Renewable materials, such as bio-based fibers and resin, offer potential environmental benefits. However, bio-composites are not implemented in the aviation industry. This is because the adaptation of new materials needs to satisfy the rigorous protection standards of aviation by reducing environmental risk, satisfying the mechanical properties, and meeting the flammability properties requirements set by the Federal Aviation Administration (FAA). Bio-phenolic is derived from cashew nut shell liquid (CNSL) which contains a phenol (carbolic acid) substitution to replace the benzene ring [1]. Additional side chain polymerization occurs between the hydroxyl group in CNSL and active methylene molecules, such as formaldehyde and hexamine [2]. Being a thermoset, phenolic is renowned for its inherent fire resistance. It is a special polymer used in electrical, automotive, and aircraft applications for its thermal stability, low smoke and flammability, and high char yield. Nevertheless, it exhibits relatively low mechanical strength and brittleness.

Therefore, a low content of multi-walled carbon nanotubes (MWCNT) as an inorganic nanofiller is used as a modifier to toughen the phenolic composites. Even though nanofillers, such as graphene [3,4] and nanoclay [5,6], have superior properties, MWCNT is chosen because of its comparable properties, lower cost, flexible processing, and ease of fabrication due to its compatibility with polymer matrix, integrating low weight and large surface area with outstanding specific strength, thermal conductivity, and electrical conductivity [7,8]. Therefore, the use of MWCNT as a reinforcing agent in polymeric composites has garnered considerable interest with new structural concepts in the composite field. Morphology and interfacial contact mechanism between MWCNTs and phenolic resin relies on the interaction of compatibilizer functional groups with carboxyl or amine groups in MWCNT [9]. Therefore, a catalytic effect is formed on polymer cross-linking that modifies the structure of the final network of polymers. Researchers explained that the enhancement in phenolic nanocomposite establishes intercalation [10] or exfoliation [11]. Furthermore, hydrogen bonds between the carbon nanotubes and the polymer chains enhance MWCNT dispersion in the polymeric matrix [12]. Therefore, the incorporation of MWCNT in polymer composites is heavily reliant on their uniform dispersion in the host matrix, which is crucial for optimizing the composite’s properties [13]. In general, the dispersion of MWCNT in the polymeric matrix is governed by two factors: (1) the morphology of MWCNT and (2) the attractive force of the tabular structure, including wall and side-wall surface [14]. Due to the smaller diameter size, MWCNT provides a large reactive surface area ratio, resulting in a high surface energy. Thus, it increases the tendency to attract nanofillers rather than the polymer matrix, causing entanglement. On the other side, attractive Van der Waals interaction between the MWCNT surfaces disrupts dispersion, may result in an inhomogeneous distribution of nanotubes. Highly inhomogeneous nanocomposites and unequal stress distribution of the applied force between non-agglomerated and agglomerated MWCNT results in the reduction of the interfacial adhesion. In general, the dispersion and distribution of MWCNTs in a polymeric matrix can be performed using a few techniques, e.g., mechanical, chemical, or a mixture of both.

MWCNT-phenolic composites can be prepared using wet or dry dispersion techniques. For the wet method, MWCNT and phenolic resin (Novolac) are dispersed in acidic solvent via ultrasonication method [15]. For the dry process, MWCNT and phenolic resin are combined without a solvent by dry ball milling. A previous study reported that a dry-mixed dispersion of 2 volume % of MWCNT in a Novolac-type phenolic resin after 25 h of ball milling exhibited a substantial improvement in flexural strength compared to a wet-mixed dispersion [16]. However, wet ultrasonication is restricted to soluble and low viscous polymers [17] and it induces modifications in structural changes to the polymer, which may weaken the composite’s mechanical and physical properties [18]. Eslami et al. reported that the flexural strength and thermal stability of phenolic composites increase with the increase in MWCNT up to 0.5 wt.% and decrease at 2 wt.% [19]. Chaiwan and Pumchusak found that 0.5 wt.% of MWCNT-reinforced phenolic resin provided the highest mechanical properties in terms of flexural and tensile strength by using a dry dispersion method [20].

*Cyrtostachys renda* (CR) from Arecaceae family are potential plant fibers that are used as reinforcement in polymer composites to add value to the resources of agro-waste. In a previous study, it has been experimentally found that bio-phenolic (Novolac) derived from CNSL is more compatible with CR fiber with a size less than 0.3 mm to enhance the mechanical properties [21]. In this research, the effect of MWCNT as a nanofiller was added to investigate the mechanical properties and the results were compared with a previous study without the presence of MWCNT-reinforced bio-phenolic composites. Synergism is observed in some cases where the matrix and reinforcing agent complemented each other with their exceptional properties. Because of the nanoscale of the nanofiller, even a small volume of reinforcing agent has a large effect on the macroscopic properties, which can have significant impact on versatility and multifunctionality of the bio-composite. Therefore, nanoscale MWCNT was applied to the micro-scale CR fiber to enhance the mechanical and flammability properties.

There are several studies that investigated on MWCNT-phenolic [19,22] and natural fiber reinforcement for phenolic composites [23,24]. Nonetheless, there is a scarcity of studies assessing the combination of MWCNT with natural-fiber-reinforced phenolic composites. Therefore, this study aims to develop bio-composites based on bio-fiber and resin, with the addition of a small amount of synthetic nano-filler that offer promising characteristics for the aviation industry. In this study, multi-walled carbon nanotubes (MWCNT) were added to phenolic resin with CR fiber and loading to investigate the physical (density, void content, water absorption, thickness swelling), mechanical (tensile, flexural, and impact), and flammability (Underwriters Laboratories test standard and limiting oxygen index) properties, and also characterized by field emission scanning electron microscopy (FESEM).This study applied the dry dispersion method for the mixing of MWCNT and phenolic resin using ball milling, while the CR fiber was mixed manually to maintain its physical structure. In addition, the properties of CR fiber reinforced MWCNT-phenolic composites are compared to those reported in a previous study without the presence of MWCNT. The development of bio-based CR fiber and phenolic resin, instead of fossil-based products, presents a feasible mitigation alternative in an attempt to lessen the environmental impacts of the aviation and automotive sectors.

## 2. Materials and Methods

### 2.1. Materials

Chemovate Girinagar, India, supplied Novolac-type dry phenolic resin. This resin is typically reddish-brown in color, with a hexamine concentration of 10% and a sieve size of 200–300 mesh. MWCNT was synthesized by using a chemical vapor deposition (CVD) process, procured from ZKK Sdn Bhd, Selangor, Malaysia, with purity exceeding 97%. The material had 8 to 15 nanotube layers with diameters ranging from 12 to 15 nm and lengths ranging from 3 to 15 μm. R&M Chemicals (Chandigarh, India) supplied the sodium hydroxide pellet, while CR fiber was extracted manually by retting from the leaf stalk sourced from a plantation in Telok Panglima Garang, Selangor, Malaysia. CR fibers were then treated for 1 h with 3% NaOH solution at an ambient temperature (24 °C). The densities of NaOH treated CR, phenolic resin, and MWCNT-phenolic were measured using a gas pycnometer and the values were 1.50 g/cm^3^, 1.30 g/cm^3^, and 1.29 g/cm^3^, respectively.

### 2.2. Fabrication of Polymer Composites

**Figure 1:**
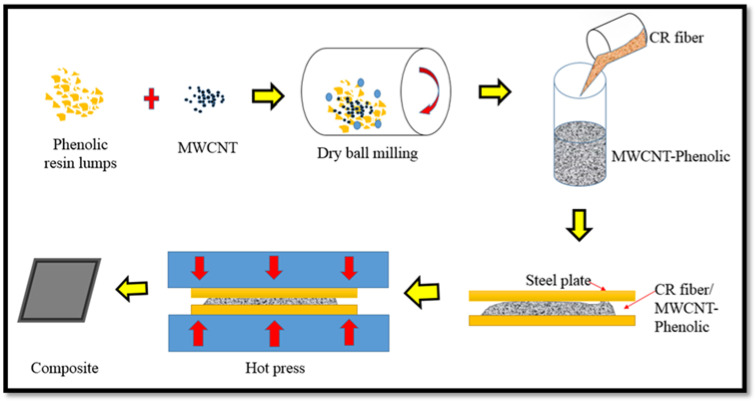
Schematic diagram of the fabrication of CR fiber-reinforcement MWCNT-Phenolic composites by dry dispersion method.

Briefly, 0.5 wt.% of MWCNT was added to phenolic resin, and the contents were mixed and milled for 25 h using a dry ball mill to achieve a homogenous distribution. The excessive amount of MWCNT is easily agglomerated, and it is difficult to disperse them uniformly in the phenolic resin. A higher loading of MWCNT results in high resin viscosity, which could affect the distribution of the CR fiber during the hot press manufacturing process. Therefore, 0.5 wt.% of MWCNT was maintained throughout the experiment. It helps to reduce the size and number of MWCNT agglomeration during the ball milling process to disperse and blend with the phenolic resin. Figure 1 depicts the fabrication process and schematic representation. The CR fiber loading was added and mixed manually with a mixture of MWCNT/phenolic according to the formulation in Table 1. The composite sheets with a thickness of 3 mm were formed by hot compressing in a steel mould for 10 min at a pressure of 10 MPa and temperature of 150 °C, respectively. The test samples were cut from the composite sheets in accordance with the specifications.

### 2.3. Characterization

#### 2.3.1. Physical Properties

##### Density and Void Content

The density of the composites was evaluated as per ASTM D1895. Five replicates of a rectangular sample with dimensions of 10 mm × 10 mm × 3 mm were prepared for each set. Each sample’s density was determined using Equation (1).

(1) $$\mathrm{Density}\;\left(g/{\mathrm{cm}}^{3}\right)=\frac{\mathrm{Mass},\;m}{\mathrm{Volume},\;v}$$

where m signifies the mass of the fiber and matrix in the composite and v signifies their volume in the composite.

The void content of the composites was calculated using Equations (2) and (3) in accordance with ASTM D2734.

(2) $$\mathrm{Void}\;\mathrm{content}\;\left(\%\right)=\frac{\rho_{\mathrm{theoretical}\;-\rho \mathrm{experimental}}}{\rho_{\mathrm{theoretical}}}\times 100$$

(3) $$\rho_{\mathrm{theoretical}}=\frac{1}{\left(\frac{Wf}{\rho_{f}}+\frac{W_{m}}{\rho_{m}}\right)}$$

where, Wf and $W_{m}$ signify for the weight of fiber and matrix fractions in the composite, respectively, whereas $\rho_{f}$ and $\rho_{m}$ represent for fiber and matrix density.

##### Water Absorption and Thickness Swelling

The water absorption test was carried out on five identical composites for each set with dimensions of 10 × 10 × 3 mm^3^ in accordance with ASTM D570. All samples were oven-dried at 80 °C for 24 h before testing and stored in a desiccator. Before being immersed in distilled water at room temperature for 24 h, the samples were weighed. The samples were withdrawn from the water and wiped with a cloth after 24 h of immersion. They were weighed once more. Weighing of samples took place for 14 days at 24 h immersion intervals. Percentage of water absorption was calculated using Equation (4),

(4) $$\mathrm{Water}\;\mathrm{absorption}\;\left(\%\right)=\frac{W_{1\;}–\;W_{0}}{W_{0}}\times 100$$

where $w_{1\;}$signifies the weight after immersion and $w_{0}$ signifies the weight before immersion.

The thickness swelling of the samples utilized in the water absorption test was evaluated. Using slide callipers, four initial thickness measurements were taken prior to sample immersion. After immersion for 24 h in the water, the samples were wiped with a clean cloth. They were dried further, and the thickness was measured again at the same four points. The process was repeated for 14 days. The thickness swelling of the composite sample was calculated using Equation (5),

(5) $$\mathrm{Thickness}\;\mathrm{swelling}\;\left(\%\right)=\frac{T_{1}\;–\;T_{0}}{T_{0}}\times 100$$

where $T_{0}$ signifies the original thickness of the sample and $T_{1\;}$ denotes the thickness after immersion.

#### 2.3.2. Mechanical Properties

##### Tensile Strength

Tensile strength tests were conducted on CR fiber-reinforced MWCNT-phenolic composites using the universal testing machine INSTRON 5566 (Instron, Norwood, MA, USA). The test was carried-out in accordance with ASTM D3039. The samples were cut into rectangular strips with dimensions of 120 × 20 × 3 mm^3^ using a band saw. The samples were prepared as rectangular strips with a dimension of 120 × 20 × 3 mm^3^ using a band saw. The sample gauge length and crosshead speed were maintained at 50 mm and 2 mm/min, respectively with a 5 kN load cell. The mean tensile strength and modulus values corresponding to the five replicates were recorded.

##### Flexural Strength

Flexural tests were performed in accordance with ASTM D7264 using INSTRON 5556 (Instron, Norwood, MA, USA). The sample was produced in a rectangular form having dimensions of 127 × 12.7 × 3.2 mm^3^ with a standard span to thickness ratio of 16:1. Cross-head speed was maintained at 2 mm/min, using a 5 kN load cell. The ASTM D7264 flexural test was performed using INSTRON 5556. The mean value of flexural strength and modulus for the five identical samples were determined.

##### Impact

Charpy impact testing was performed on composite samples in accordance with ASTM D256 using the Gotech GT-705-MD model. Five identical samples with dimensions of 64 × 12.7 × 3 mm^3^ were prepared and tested for each set, and the average impact strengths were calculated using Equation (6).

(6) $$\mathrm{Impact}\;\mathrm{strength}\;=\frac{\mathrm{Impact}\;\mathrm{energy}\;\left(\mathrm{kJ}\right)}{\mathrm{Area}\;\left(m^{2}\right)}$$

#### 2.3.3. Morphology

The morphology of the tensile fracture surface was examined using a Nova NanoSEM 230 Series Field Emission Scanning Electron Microscope (FESEM) (FEI, Hillsboro, OR, USA) to examine the dispersion and aggregation of the CR fiber and MWCNT in the phenolic composites. All the samples were sputter-coated with platinum to prevent the charge effect.

#### 2.3.4. Flammability Properties

Underwriters Laboratories (UL 94) and the limiting oxygen index (LOI) were used to determine the flammability properties of the CR fiber-reinforced MWCNT-phenolic composites.

##### Underwriters Laboratories (UL 94)

UL 94 assesses the plastic material’s responsiveness to a radiant heat source used to extinguish a fire upon ignition, as well as its dripping performance under controlled conditions. The flammability of the phenolic composites was assessed using horizontal burning test as per ASTM D 635. For each set, five identical rectangular samples with dimensions of 125 × 13 × 3 mm^3^, were prepared. Then, 25 mm from either edge of the long sides was marked. A flame was applied to one end of the specimen after it was positioned horizontally. The time it takes for the flame to move from 25 mm (from the end) to 100 mm was recorded. The burning rates of the composites were then estimated using Equation (7).

(7) Burning velocity, V=60 L/t

where *V* denotes the burning velocity rates (mm/min), *L* the burnt length (mm), and *t* the burning time (s).

##### Limiting Oxygen Index (LOI)

The limiting oxygen index (LOI) is the minimal concentration of oxygen required for ignition, given as a volume percentage. The ten identical test specimens with a sample size of 100 mm × 6.5 mm × 3 mm were prepared to perform the LOI test in accordance with ASTM D 2863. The specimen was clamped vertically in the sample holder and allowed to burn for ten seconds at a time until ignition ignited. The LOI was calculated using Equation (8).

(8) $$\mathrm{LOI}\;\left(\%\right)=\frac{O_{2}}{\left(O_{2}+N_{2}\right)}\times 100$$

where $O_{2}$ represents the volumetric flow rate of oxygen (cm^3^/s) and $N_{2}$ the volumetric flow rate of nitrogen (cm^3^/s).

### 2.4. Technique for Order Preference by Similarity to the Ideal Solution (TOPSIS)

The technique for order preference by similarity to the ideal solution (TOPSIS) method is used in order to find an optimal configuration of phenolic composites for mechanical and flammability properties. The TOPSIS method takes into consideration both the degree to which each alternative deviates from positive and negative ideals. There are four criteria and five alternatives in this study with their weightage as presented in Table 2. There are a few steps to be followed in order to obtain relative closeness as described in [25].

## 3. Results and Discussion

### 3.1. Physical Properties of CR Fiber-Reinforced MWCNT-Phenolic Composites

The density and void content of phenolic (PH), MWCNT/PH, and CR/MWCNT/PH with various loadings of the CR fiber are given in Table 3. The table displays that the addition of the CR fiber slightly increases the density of the composites as much 8% higher than the matrix’s density. Based on theoretical and experimental values, the void content was calculated. The pure phenolic resin exhibited the lowest void content, followed by the phenolic with MWCNT filler. The void is formed during the hot-pressing process where the condensation of the phenolic resin during the cross-linking reactions creates volatiles. The voids would also develop if the matrix did not fully wet out the fibers during the fabrication process [26].

**Figure 2:**
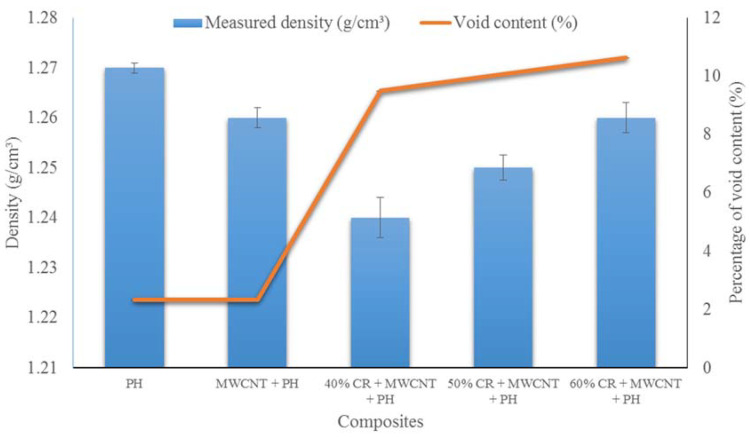
Density and voids content of CR fiber-reinforced MWCNT/phenolic composites.

The density and void content of the CR fiber-reinforced MWCNT/phenolic composites are shown in Figure 2. As the amount of CR fibers increases, the void content decreases. This could be because of the poor interfacial adhesion between the fiber and matrix, which contributes to the void content increase [27]. Based on the morphology, the CR fiber contains a hollow cellular structure or lumen similar to an oil palm empty fruit bunch fiber [28]. Due to the high viscosity of the MWCNT-phenolic fluid, the CR fiber was not thoroughly wetted, reducing the fiber–matrix interfacial strength.

**Figure 3:**
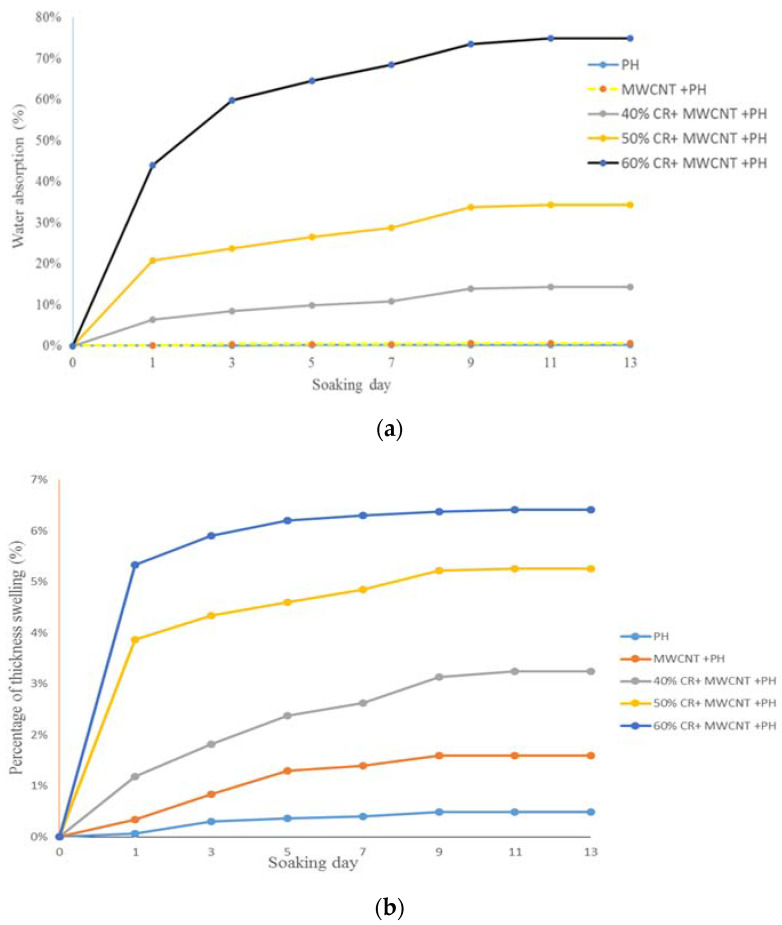
(**a**) Percentage of water absorption and (**b**) thickness swelling of CR reinforced MWCNT-Phenolic composites.

Figure 3a,b show the percentage of water absorption and thickness swelling, respectively, of all samples. Water absorption and swelling increased with increasing immersion duration until saturation was attained. The available free volume or local mobility as well as the amount of “open” hydrogen bonds in the polymer determine the saturation level of water absorption [29,30]. The lowest water absorption and thickness swelling was attained for phenolic resin, followed by MWCNT-phenolic composites. Water absorption in polymer composites could be explained by the interaction of water molecules with the polymer matrix [31] and their mobility [32], classified as type I and type II [33]. In type I, water diffuses freely across the polymer network, breaking the interchain Van der Waals and hydrogen connections and promoting chain segmental mobility and polymer plasticization [31]. Whereas in type II, water is coupled with or “bonded” to water molecules that establish many hydrogen bonds with the polymeric network and contribute little to the plasticization process [33]. Nonetheless, the formation of secondary crosslinking arises as a consequence of the creation of bridges across structural segments. The water absorption properties of phenolic and MWCNT-phenolic are known to be type II, which is associated with water molecules trapped in micro voids or clustered water.

**Figure 4:**
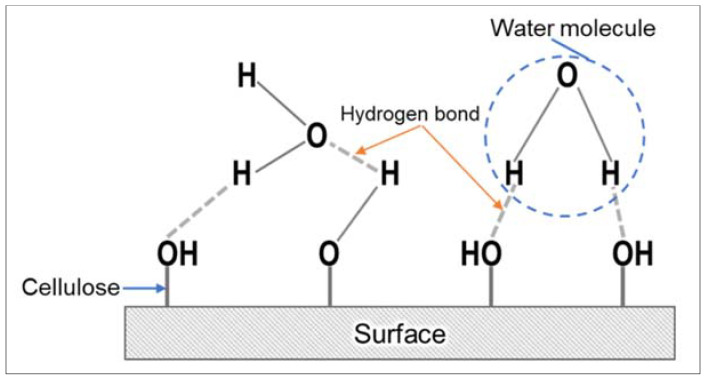
Diffusion of water molecules into CR fiber-reinforced MWCNT-phenolic composites.

**Figure 5:**
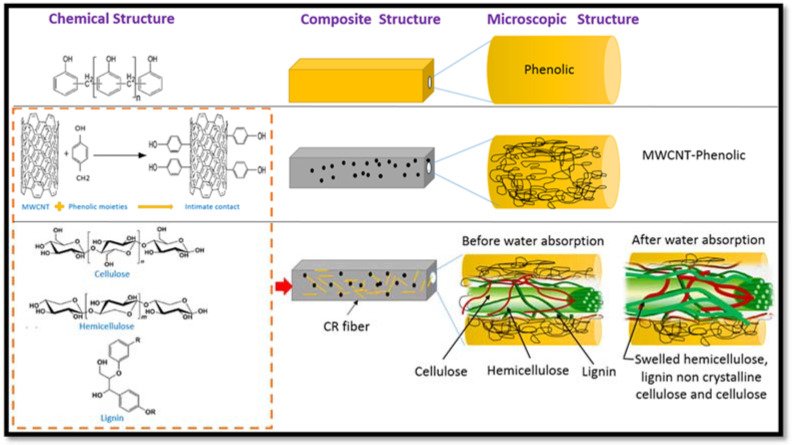
Schematic diagram of water absorption of CR fiber-reinforced MWCNT-Phenolic composites.

The addition of CR fiber showed a higher water absorption and thickness swelling than the phenolic resin and MWCNT-phenolic composites. The loading of CR fibers is proportional to the absorption of water and the thickness swelling. The cellulose content of CR fibers, which are hydrophilic and hygroscopic, are attributed to higher water absorption and thickness swelling. This is most likely because the hydrogen interaction between the water molecules and the free hydroxyl groups present in the CR cellulosic wall and MWCNT diffused the water molecules into the CR fiber and matrix interface, as seen in Figure 4. Water molecules penetrate by capillary forces between the fibers and interfere with the cellulose [34]. These –OH groups attract water molecules and make hydrogen attachments with them, which attract cellulose molecules into the H_2_O molecules. The water molecules are often drawn to each other. Hence, the cellulose fibers take in huge quantities of water. Therefore, as CR loading increases, it leads to the formation of more water resident sites (–OH groups), resulting in an increase in water absorption rate, as illustrated in Figure 5. However, as the layer of hemicellulose was removed, the crystallinity of NaOH-treated CR fiber increased as reported in [35]. This limits the rate of water absorption compared to untreated fiber because the hydroxyl groups in hemicellulose, responsible for engaging the water molecules, are removed.

Table 4 presents the comparison of CR fiber-reinforced phenolic composites with and without the presence of MWCNT. The presence of MWCNT in phenolic resin and reinforcement with CR fiber with a loading of 40 wt.% showed an increment in the water absorption and reduction in the thickness swelling with respect to the CR-reinforced phenolic resin [21]. The higher water absorption of the CR fiber-reinforced MWCNT-phenolic composites were due to the presence of –OH and –COOH hydrophilic groups on the MWCNT [36],which further increased the water absorption in CR fiber-reinforced phenolic composite. MWCNTs also act as additional crosslinks, increasing the crosslinking density of the polymeric matrix by reducing free volume due to the segmental immobilisation of polymer chains [33]. As a result, the thickness swelling of 40% of the CR fiber-reinforced MWCNT-phenolic composites was lower compared to 40% of the CR fiber-reinforced phenolic composites. Therefore, the incorporation of MWCNT in the phenolic resin improved the dimensional stability.

### 3.2. Mechanical Properties of Polymer Composites

#### 3.2.1. Tensile Strength

**Figure 6:**
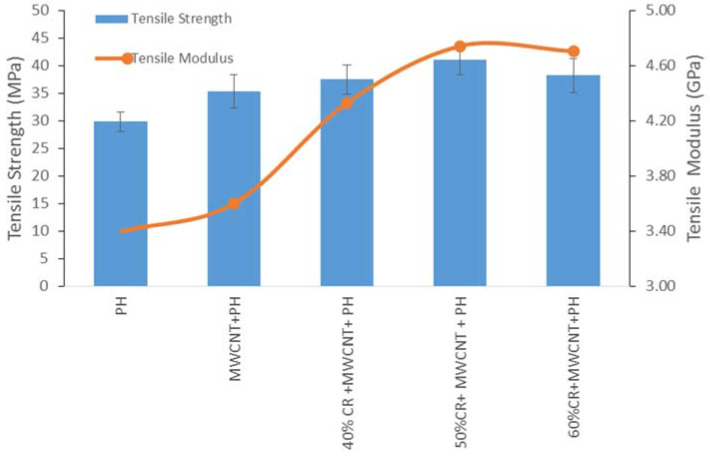
CR fiber loading on tensile strength and Modulus of MWCNT-Phenolic composites.

**Figure 7:**
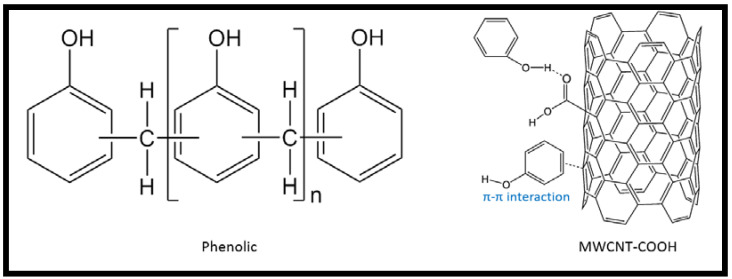
Chemical structure and bonding of MWCNT-Phenolic, reproduced from Ref. [50] with permission from the Royal Society of Chemistry.

The tensile strength and the effect of the CR fiber loading in MWCNT-phenolic composites is illustrated in Figure 6. The tensile strength and tensile modulus of the phenolic composite improved the tensile strength and modulus by as much as 5.5% and 6%, respectively, with the addition of 0.5 wt.% of MWCNT. A similar results was found in [37,38], where the addition of MWCNT improved the tensile properties especially the strength and modulus of polymeric composites. Yeh et al., who investigated the tensile properties of 0.5 wt.% MWCNT-phenolic composites, found the similar results, with the tensile strength, Young’s modulus increased compared to pristine phenolic [39]. The characteristics of composites are modified by the dispersion and distribution of MWCNT in the polymeric matrix. Effective dispersion and distribution of MWCNT in composites minimizes stress concentration and improves the uniformity of stress distribution. Therefore, the tensile properties improved [40]. The interaction between MWCNT and phenolic composites could be explained in mechanical and chemical terms. In the mechanical aspect, the role of MWCNT in the phenolic resin for the tensile properties could be explained in two modes. First, MWCNT’s crystalline nature [41] increases the crystallinity index, which improves the tensile properties. The increase in crystallinity influences the interfacial adhesion between the MWCNT surfaces and the phenolic bonding. Thus, this restricts the polymer matrix’s segmental mobility in the elastic zone, hence improving the modulus [37]. Second, the higher surface energy results in a strong interfacial adhesion and linkage between the MWCNT surfaces and the phenolic structure, facilitating more efficient load transfer from the matrix to the nanotubes [40]. According to Wagner et al., the stress transferral performance of multi-walled nanotubes/polymers is at least one order of magnitude higher than the typical fiber-based polymeric composites [42]. However, based the chemical interaction of MWCNT and phenolic resin also enhanced the interfacial bonding. The presence of carboxyl and hydroxyl functional groups in MWCNTs strengthens the interfacial bonding between MWCNT and polymeric matrix [43]. Botelho et al. supported that strong hydrogen interaction between the hydroxyl groups of the MWCNTs and the matrix allows the MWCNT dispersed in the phenolic resin [44] to be bonded covalently [15]. According to Taherian et al., the existence of –COOH peaks from the Fourier transform infrared spectroscopy study is an indication of the bonding and crosslinking formation between the MWCNT and the phenolic resin [18] as shown in Figure 7.

When the CR fiber was added as much as 40 wt.%, the tensile strength and modulus of the MWCNT-phenolic composites improved as much as 6% and 18%, respectively. Further addition of 50 wt.% of CR fiber provided the highest tensile strength and modulus properties for MWCNT-phenolic composites. This is because higher fiber content offers an increased load carrying capacity in composites [45,46]. As a result, the fiber bears more load than the matrix if the composite is loaded with certain strain. The decline in tensile strength and modulus occurred as the CR fiber was increased to 60 wt.%. This is attributed to excessive fibers in the matrix, resulting in inadequate wettability of the fiber by the matrix, hence reducing the fiber-matrix interfacial strength. According to Chen et al., fiber wettability is closely related to fiber surface free energy, which is determined by the functional groups and polarity of the fiber [47]. The higher fiber loading resulted in fiber entanglement rather than fiber dispersion and distribution in the polymeric composites. Agglomeration and increased viscosity of short fibers in randomly oriented composites result in reduced tensile strength and strain at composite fracture [48].

In previous study, 40 wt.% CR fiber loading without MWCNT, the tensile strength and modulus were reported to be 34.27 MPa and 3.77 GPa, respectively as reported in [21]. The addition of MWCNT in 40 wt.% CR fiber-reinforced phenolic composite enhanced the tensile strength and modulus around by 10% to 15%, respectively. It is indicated that MWCNT incorporation with CR fiber-reinforced phenolic composites enhance the tensile properties by creating interlocking between CR fiber and phenolic matrix. Similar findings have been reported by [16,49], who found MWCNT to be strengthening the interface of the fiber–matrix. The addition of 50 wt.% CR fiber and MWCNT in the phenolic composite improved the tensile strength and modulus by about 38% in comparison to the pristine phenolic.

#### 3.2.2. Morphology

**Figure 8:**
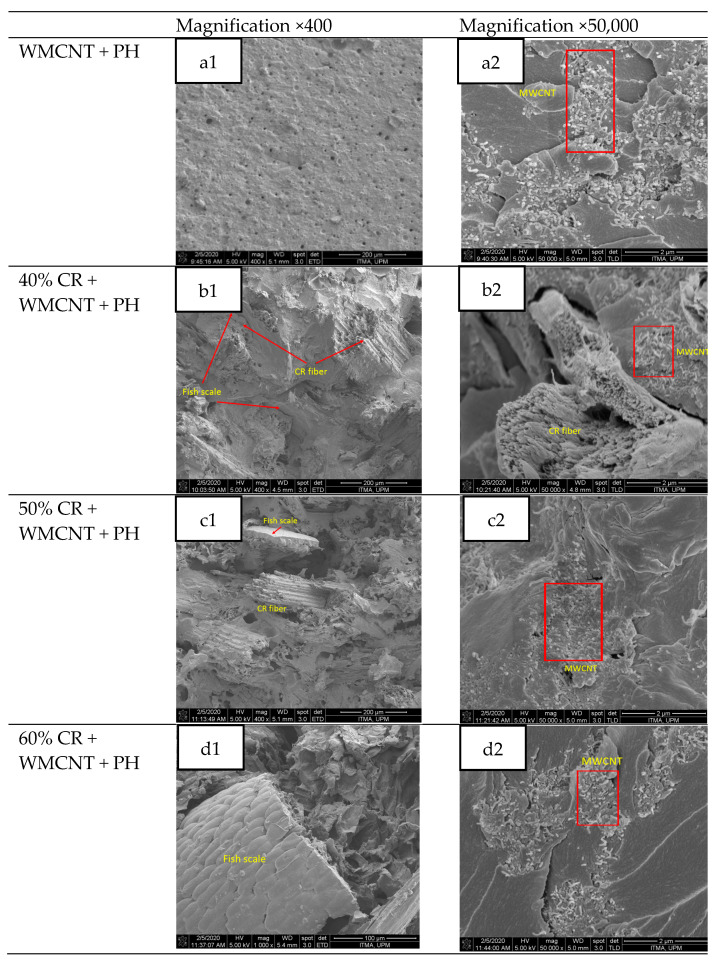
FESEM micrographs of the bio-composites filled with MWCNT and CR fiber (**a1**,**a2**) MWCNT + PH, (**b1**,**b2**) 40% CR + MWCNT + PH, (**c1**,**c2**) 50% CR + MWCNT + PH, (**c1**,**c2**) 50% CR + MWCNT + PH, (**d1**,**d2**) 60% CR + MWCNT + PH.

The influence of MWCNT on the tensile properties of the bio-composites was supported by the FESEM micrographs. In this section, the dispersion of MWCNT in CR/phenolic resin bio-composites is examined as shown in Figure 8 with a magnification of 400 and 50,000 to illustrate the bonding of the CR fiber and the dispersion of MWCNT, respectively. A phenolic resin contains the MWCNT in which the MWCNTs are covalently bound to the matrix by the phenolic moieties [15]. In the cases of nanocomposites, the dispersion methods, and the interactions between the phenolic matrix and the MWCNT, the microstructure of nanocomposites is described as intercalated. The dispersion and intercalation of MWCNT in a phenolic matrix was observed to be in a crystallographically irregular order as depicted in Figure 7. This is also supported by [51] because the forces holding the stacks together are weak through Van der Waals interactions, nanoparticles are intercalated between the phenolic resin. The images also revealed that the inclusion of MWCNT in phenolic composites exhibited a good distribution but a poor dispersion quality with noticeable agglomeration. The MWCNT nano particles appeared to aggregate and agglomerate as well as shape a micron filler cluster, restricting the uniform dispersion of MWCNT in the phenolic matrix [52].

In Figure 8(b1–d1), showing the composites at a magnification of 400×, the appearance of fish scale depicts that the fiber strongly adhered to the matrix. As lignocellulosic fibers were used as a reinforcing agent of a phenolic resin [53,54], the issue of a lack of adhesion was reduced as specific associations were formed between the polar groups of fibers and the phenolic matrix [55]. The existence of polar groups in the phenolic matrix’s chemical structure resulted in the interaction between the lignocellulosic fibers and was an advantage compared to hydrophobic matrices. The emergence of the fish scale implies a high degree of compatibility between the fibers and matrix, resulting in a cohesive interface.

Figure 8b1,c1 illustrate the CR fiber pull-out in MWCNT-phenolic composite. The surface area contact was more extensive in a shorter length of the fiber, which led to stronger interfacial bonding. The loads of fiber pull-out increased as the fiber content increase. This increase in the tensile strength of 50 wt.% fiber corresponds to an increase in pull-out load. It is associated with the increase in the weight fraction of the fiber, which results in increased strength of the fiber composite ligament.

#### 3.2.3. Flexural Strength

**Figure 9:**
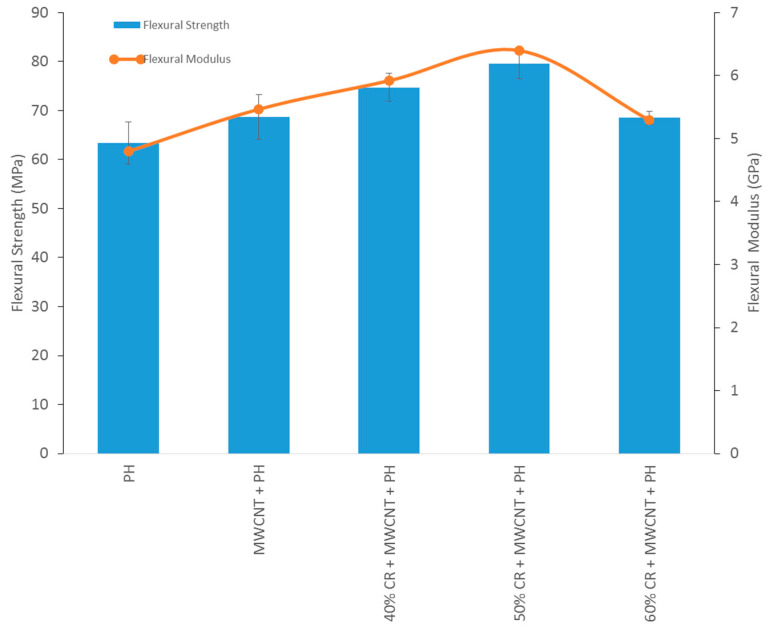
Effect of CR fiber loading on flexural strength and Modulus of MWCNT-Phenolic composites.

The flexural strength and modulus of neat phenolic, MWCNT-phenolic, and fiber loadings of CR on MWCNT-phenolic composites are presented in Figure 9. The flexural strength and modulus of the phenolic resin increased to 8% and 14%, respectively, with the incorporation of MWCNT. In this case, the lower content of MWCNT improved the flexural properties, as indicated by researchers [19], who reported that the flexural characteristics of nanocomposites improved as MWCNT content (wt.% ≤ 0.5), but declined as MWCNT content exceeded 2 wt.%. This is because MWCNT acts as a reinforcement by strengthening the fiber-matrix interface. It is also supported by Yeh et al. that an improved wetting between the MWCNT and the phenolic resin is owing to the fact that the two materials have a higher interfacial area [39]. A similar finding from [49] showed that well-dispersed carbon nanotubes that bridged the interface micro-cracks led to an enhancement in the fiber–matrix interface and subsequent improvement in flexural properties. The role of MWCNT in the phenolic composites, in the chemistry aspect, is based on their covalent bonding and the formation of crosslinking MWCNT and phenolic resin as explained in the case of tensile properties.

In comparison to MWCNT-phenolic composites, the addition of 40 wt.% CR fiber enhanced flexural strength and modulus about 8%. Further addition CR fiber to 50 wt.%, significantly improved the flexural strength and modulus by as much as 15% and 17%, respectively. The MWCNT-phenolic resin incorporated with a low fiber loading increases the mechanical characteristic of the composite materials, but not at the optimum level. The high fiber loading contributed to a high load transfer ability of the fiber [56]. However, further addition of the fiber loading up to 60 wt.% led to a reduction in the flexural properties. This may be due to the presence of starvation resin [57] from a higher fiber contact causing the incomplete impregnation of the fibers by the polymeric matrix. This indicates that the addition of CR fiber and intercalation of MWCNT in the phenolic composites improved the flexural properties.

In a previous study, using 40 wt.% CR fiber loading without MWCNT, the flexural strength and modulus were reported to be 65.26 MPa and 5.80 GPa, respectively [21]. The addition of MWCNT in 40 wt.% CR fiber-reinforced phenolic composite improved the flexural strength and modulus by around 14% and 2%, respectively. It is indicated that MWCNT incorporation with CR fiber-reinforced phenolic composites increase the flexural properties by creating interlocking between CR fiber and phenolic matrix, as described previously.

#### 3.2.4. Impact Strength

**Figure 10:**
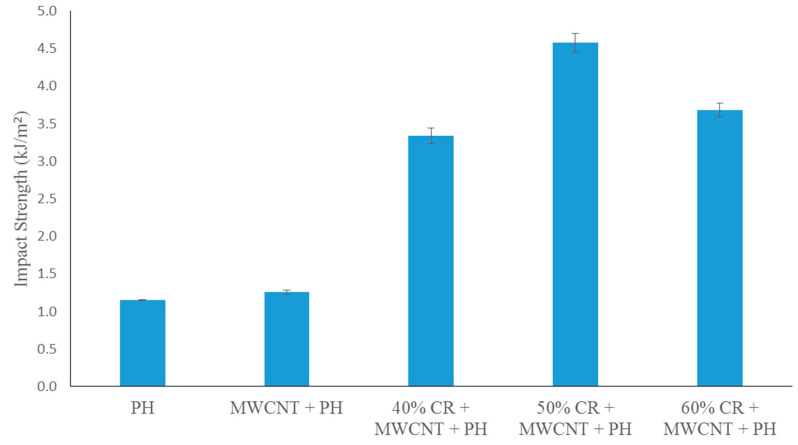
Effect of the fiber loading on impact strength of MWCNT-Phenolic composites.

Charpy impact testing was performed to investigate the impact of energy absorption capability by the effect of CR fiber loading and addition of MWCNT filler in the phenolic composites. The impact strength values denote the total capacity of the absorbed energy needed to ultimately fracture the specimen. The results from the Charpy impact test of the CR fiber loading on the MWCNT-phenolic composites are shown in Figure 10. The phenolic resin showed a low impact strength owing to its high cross-linking structure.

The incorporation of 0.5 wt% of MWCNT in the phenolic composite slightly improved the impact strength to approximately 8%. Similar finding also found by Edson et al., who investigated the impact strength of CNT-phenolic, where CNT offered higher impact energy than the pristine phenolic [42]. The higher impact energy is attributed to capacity of MWCNT to allow more impact load transfer from the matrix. In this case, MWCNT acts as a medium of load transferral.

The addition of CR fiber has resulted in a remarkable enhancement in the impact strength of the composites in comparison to the neat phenolic and MWCNT-phenolic composite. The addition of 40 wt.% of CR fiber improved the impact strength as much as 167% compared to MWCNT-phenolic composite. Meanwhile, the addition of 50 wt.% of CR fiber provides optimal impact strength by improving the 266% impact strength of MWCNT-phenolic. Similar findings have been reported by [23,58,59], who found that the incorporation of natural fibers in the phenolic composites provides higher impact energy.

**Figure 11:**
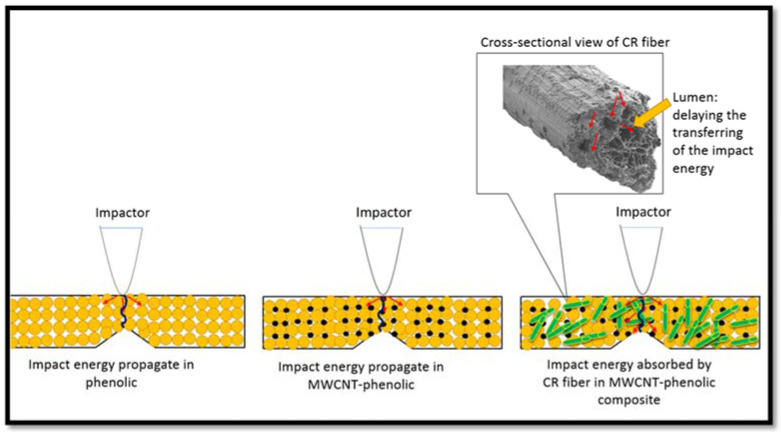
Schematic diagram of the impact energy propagation in CR fiber-reinforced MWCNT-Phenolic composites.

This is attributable to the bridge linkage mechanism [60,61], fiber pull-out, and fiber fracture-absorbing additional energy, which inhibit crack proliferation and improve the composite’s toughness [62]. Ramires et al. studied the impact strength on sisal fiber-reinforced tannin-phenolic resins and found that the composites revealed the mechanism of fiber-bridging [61]. Not all fibers were fractured during the impact in this mechanism, as some fibers continued to form a “bridge” between the two parts of the fractured matrix. In this case, the CR fibers were acting as an energy absorber. The CR fiber has a hollow brick-wall structure as revealed by SEM micrograph [63]. When the load is transferred from the matrix to the fiber, the hollow brick-wall structure and lumen of the CR fiber slows down or delays the impact force propagation when passed through it, resulting in a reduction in the force momentum. Hence, the impact strength of the composites is increased as illustrated in Figure 11. However, when the CR loading reached about 60 wt.%, it reduced the impact strength of the composites. This may be owing to the non-homogenous distribution of the fiber and non-intense interaction of the interface between the fiber and the matrix, leading to the weak impregnation of the fiber by the matrix.

In a previous study, using 40 wt.% CR fiber loading without MWCNT, the impact strength was found to be 2.03 kJ/m^2^ [21]. However, the addition of MWCNT in 40 wt.% CR fiber-reinforced phenolic composite enhanced the impact strength by as much as 65%. It is indicated that MWCNT well incorporated with CR fiber-reinforced phenolic composites to enhance the impact properties by creating interlocking between CR fiber and phenolic matrix, as described previously.

### 3.3. Flammability Properties of Polymer Composites

#### 3.3.1. Underwriters Laboratories Test Standard (UL94 Horizontal Burning Test)

A common method for the determination of the relative flammability for plastic materials is the UL 94 horizontal test. This method analyzes a controlled flame ignition source for a predetermined amount of time to determine the specimen’s burning rate in mm/min. For all sets of samples, the burning was stopped before marking. As a result, all composite samples classified as H-B (horizontal burn) were categorized as self-extinguishing materials, as tabulated in Table 4. As can be seen, the UL94 horizontal flammability of the MWCNT-phenolic composites with natural fiber addition demonstrated good results, despite the fact that natural fibers are very flammable. The presence of a higher lignin concentration in the CR fiber [35] resulted in the production of char, which improved the composites’ flammability properties [64]. Char development during combustion of the MWCNT-phenolic composite-based CR fiber resulted in the cessation of further combustion heat flow. Char acts as a physical and diffusion barrier of heat flux and fuel flux, respectively, from the polymer [65]. Char formed solely in a cross-linked polymer contains aromatic fragments which can be aromatized during thermal decomposition [66]. The burning rate of the horizontal burn for all the samples was 0, inferring all the samples as self-extinguishing material.

#### 3.3.2. Limiting Oxygen Index (LOI)

The LOI reading is a comparison of materials in which a higher oxygen index indicates lower flammability. Thus, the LOIs of MWCNT-phenolic and CR fiber-reinforced MWCNT-phenolic composites were measured to determine their flame retardant properties. The impact of MWCNT and CR fiber on the flammability of phenolic composites can be determined using the LOI test, as indicated in Table 5. The pristine phenolic LOI value of 29.33 indicates that it is a good flame retardant, mainly due to the considerable number of benzene rings present in the phenol structure. Phenolic resin resulted in a charring coating on the burnt residue during combustion, which would avoid oxygen dissemination. This char formation serves as a thermal shield between the heat source and the lower polymeric layers, thus increasing the value of LOI [67]. However, the LOI values decreased with the addition of MWCNT and CR fiber. When the MWCNT-phenolic composite was exposed to the flame, it began to decompose into different volatile gases and char was formed. The volatile gases react with the ambient oxygen and intensify the burning process, which was particularly visible around the specimen’s edge. The flammability properties are interlinked with the viscosity of the polymer [68]. When MWCNT was filled with the phenolic resin, the mobility of the polymeric chain segment was hindered. Hence, the viscosity of the polymer increased [69]. The LOI ratio is the highest for the pristine phenolic when it was burned in a small fire. However, the MWCNT-phenolic burning requires more effective combustion owing to the development of a network structure of the char layer which may block the polymer composites and increase viscosity, resulting in a decrease in LOI [70]. In the previous studies, the researchers found that adding boron at 3.4 wt.% increased the LOI value to 38% [71], while adding Aluminum Diethylphosphinate at 10 wt.% and 15 wt.% increased the value to 33.1% and 34.1% [72], respectively. Due to the lower concentration of MWCNT (0.5 wt.%) added to the phenolic in this research, the quantity of char produced by MWCNT considered low, implying a lower LOI value than other flame additives.

In a previous study, using 40 wt.% CR fiber loading without MWCNT, the LOI value was reported to be 25.41% [21],however the addition of MWCNT improved to 26.79%. This is because MWCNT functioned as a flame retardant mechanism by forming char layers [73]. Because MWCNTs are evenly dispersed in the phenolic resin, after burning the composites, the carbon forms a thin network that prevents oxygen and heat from contacting the next composites and increases the flame retardation [74]. In addition, MWCNT percolates to form a network in the CR fiber-reinforced phenolic resin, leading to flame retardant properties [75]. A similar finding by Toldy et al. showed that, with regard to pristine epoxy, the carbon nanotubes in epoxy composites slightly decreased the LOI value [76]. Even the LOI value of addition of MWCNT in phenolic reduced slightly. However, the combination of MWCNT in CR fiber reinforced phenolic composites enhanced the flammability resistance. The LOI value decreases with the increase in the CR fiber loading, as CR fibers are considered flammable materials. However, the LOI value exceeding 25% is regarded in the category of flame retardant and self-extinguishing material [77]. These flammability tests comply with the Federal Aviation Regulation (FAR) requirement. The hemicellulose content in the natural fiber is responsible for the thermal stability and flammability. However, lignin contributes to char formation. The 3% NaOH-treated CR fiber reduced the content of hemicellulose by 17% and increased the lignin content by 10% as reported in [35].

### 3.4. TOPSIS Method

The TOPSIS approach was used to rank and determine the best composite characteristics for possible applications. The decision matrix, normalization matrix, weight-normalized matrix, positive and negative ideal values, distance to positive and negative ideal points, relative closeness value known as ci value, and ranking are shown in Table 6, Table 7, Table 8, Table 9 and Table 10. Table 6 depicts the decision matrix, whereas Table 7 depicts the normalized matrix. Table 8 and Table 9 illustrate the weighted normalized decision matrix as well as the positive and negative ideal values, respectively. Table 10 shows the distance to the positive and negative ideal solutions, as well as the degree of closeness of each alternative to the ideal solution.

**Figure 12:**
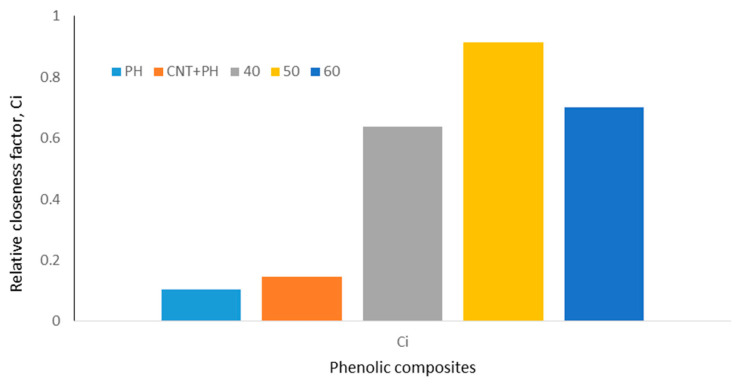
Relative closeness factor of phenolic composites.

Figure 12 depicts the ranking of composite mechanical and flammability properties according on their proportion and attributes. The optimal phenolic composite, according to the results, was a 50 wt.% CR fiber-reinforced MWCNT-phenolic composite with a relative closeness to the best solution and values of 0.913. The TOPSIS technique was determined to be the most effective way for choosing the optimum composite proportion from a set of composites [78].

## 4. Conclusions

In this study, CR fiber reinforced-phenolic nanocomposites were produced by incorporating MWCNT using a dry dispersion method, and the physical, mechanical, and flammability properties were investigated. In the CR fiber-reinforced MWCNT-phenolic composites, the void content increased as the content of CR fiber increased. The presence of MWCNT significantly reduced the thickness of the swelling of CR fiber-reinforced MWCNT-phenolic composites, thus enhancing the dimensional stability of the composites. The optimum flexural, tensile, and impact strengths of the MWCNT-phenolic composites were obtained for 50 wt.% of CR fiber incorporation. MWCNT well incorporated CR fiber-reinforced phenolic composites to improve the mechanical properties by creating interlocking between CR fiber and phenolic matrix. The FESEM results showed that the CR fiber was well adhered to the matrix and indicated that the interface between the CR fiber and the MWCNT phenolic resin is strong. The UL-94 horizontal and LOI tests showed that all the tested composite materials can classified as self-extinguishing. Based on the TOPSIS method, 50 wt.% CR fiber-reinforced phenolic composite was selected as the best composite for structural applications. These bio-based composites are low-cost and exhibit desirable mechanical and flammability properties. They are, therefore, ideal for broader applications in the transport industry.

## Figures and Tables

**Table 1:** Composites formulation.

Designation Ratio of Composites	Phenolic Resin (wt.%)	0.5 wt.% MWCNT– Phenolic (wt.%)	CR Fiber (wt.%)
PH	100	0	0
MWCNT + PH	0	100	0
40% CR + MWCNT + PH	0	60	40
50% CR + MWCNT + PH	0	50	50
60% CR + MWCNT + PH	0	40	60

CR: *Cyrtostachys renda* fiber.

**Table 2:** Characteristics of criteria.

Properties	Type	Weight
Tensile	+	0.25
Flexural	+	0.25
Impact	+	0.25
LOI	+	0.25

**Table 3:** Density and void content of CR fiber-reinforced MWCNT-phenolic composites.

Weight Fraction of Fibers (%)	Theoretical Density (g/cm^3^)	Measured Density (g/cm^3^)	Void Content (%)
PH	1.30	1.27	2.31
MWCNT +PH	1.29	1.26	2.33
40% CR + MWCNT + PH	1.37	1.24	9.49
50% CR + MWCNT + PH	1.39	1.25	10.07
60% CR + MWCNT + PH	1.41	1.25	10.63

**Table 4:** Comparison of CR fiber-reinforced MWCNT-Phenolic composites with CR fiber-reinforced phenolic composites.

Fiber	Resin	Loading	Immersion Day	Water Absorption (%)	Thickness Swelling	Ref.
CR	Phenolic	40 wt.%	1	2.41	3.87	[21]
CR	MWCNT-Phenolic	40 wt.%	1	6.40	1.18	Current Study
CR	Phenolic	40 wt.%	7	7.20	9.24	[21]
CR	MWCNT-Phenolic	40 wt.%	7	10.86	2.63	Current Study

**Table 5:** UL 94 and LOI for CR fiber-reinforced MWCNT-phenolic composites.

Composites	UL-94 Horizontal (mm/min)		LOI (%)	Ref.
	Burning Rate, V	Classifications		
PH	0	H-B	29.33	Present study
MWCNT + PH	0	H-B	27.59	Present study
40% CR + PH	0	H-B	25.41	[21]
40% CR + MWCNT + PH	0	H-B	26.79	Present study
50% CR + MWCNT + PH	0	H-B	26.32	Present study
60% CR + MWCNT + PH	0	H-B	25.67	Present study

Note: Flame is extinguished before the first mark (UL-94 Horizontal).

**Table 6:** Decision Matrix.

	Tensile	Flexural	Impact	LOI
PH	29.82	63.39	1.15	29.33
MWCNT + PH	35.34	68.67	1.25	27.59
40% CR + MWCNT + PH	37.47	74.74	3.34	26.79
50% CR + MWCNT + PH	41.06	79.64	4.58	26.32
60% CR + MWCNT + PH	38.24	68.53	3.68	25.67

**Table 7:** The normalized matrix.

	Tensile	Flexural	Impact	LOI
PH	0.365	0.398	0.165	0.483
MWCNT + PH	0.432	0.431	0.179	0.454
40% CR + MWCNT + PH	0.458	0.469	0.479	0.441
50% CR + MWCNT + PH	0.502	0.5	0.657	0.433
60% CR + MWCNT + PH	0.468	0.43	0.528	0.423

**Table 8:** The weighted normalized matrix.

	Tensile	Flexural	Impact	LOI
PH	0.091	0.100	0.041	0.121
MWCNT + PH	0.108	0.108	0.045	0.114
40% CR + MWCNT + PH	0.115	0.117	0.120	0.110
50% CR + MWCNT + PH	0.125	0.125	0.164	0.108
60% CR + MWCNT + PH	0.117	0.108	0.132	0.106

**Table 9:** The positive and negative ideal values.

	Positive Ideal	Negative Ideal
TENSILE	0.125	0.091
FLEXURAL	0.125	0.100
IMPACT	0.164	0.041
LOI	0.121	0.106

**Table 10:** Distance to positive, negative ideal points and the relative closeness value and ranking.

Composites	Distance to Positive and Negative Ideal Points		The Relative Closeness Value and Ranking	
	Distance to Positive Ideal	Distance to Negative Ideal	Relative Closeness, C_i_	Rank
PH	0.13	0.015	0.104	5
MWCNT+PH	0.122	0.021	0.145	4
40% CR + MWCNT + PH	0.048	0.084	0.638	3
50% CR + MWCNT + PH	0.012	0.13	0.913	1
60% CR + MWCNT + PH	0.041	0.095	0.700	2
